# Molecular Dynamics Simulations of Human FOXO3 Reveal Intrinsically Disordered Regions Spread Spatially by Intramolecular Electrostatic Repulsion

**DOI:** 10.3390/biom11060856

**Published:** 2021-06-08

**Authors:** Robert O.J. Weinzierl

**Affiliations:** Department of Life Sciences, Imperial College London, Sir Alexander Fleming Building, Exhibition Road, London SW7 2AZ, UK; r.weinzierl@imperial.ac.uk

**Keywords:** apoptosis, cancer, longevity, FOXO3, local compaction tool, intrinsically disordered protein, DNA-binding, transcription factor, molecular dynamics simulation, computational biology

## Abstract

The human transcription factor FOXO3 (a member of the ‘forkhead’ family of transcription factors) controls a variety of cellular functions that make it a highly relevant target for intervention in anti-cancer and anti-aging therapies. FOXO3 is a mostly intrinsically disordered protein (IDP). Absence of knowledge of its structural properties outside the DNA-binding domain constitutes a considerable obstacle to a better understanding of structure/function relationships. Here, I present extensive molecular dynamics (MD) simulation data based on implicit solvation models of the entire FOXO3/DNA complex, and accelerated MD simulations under explicit solvent conditions of a central region of particular structural interest (FOXO3^120–530^). A new graphical tool for studying and visualizing the structural diversity of IDPs, the Local Compaction Plot (LCP), is introduced. The simulations confirm the highly disordered nature of FOXO3 and distinguish various degrees of folding propensity. Unexpectedly, two ‘linker’ regions immediately adjacent to the DNA-binding domain are present in a highly extended conformation. This extended conformation is not due to their amino acid composition, but rather is caused by electrostatic repulsion of the domains connected by the linkers. FOXO3 is thus an IDP present in an unusually extended conformation to facilitate interaction with molecular interaction partners.

## 1. Introduction

The members of the vertebrate ‘forkhead box O’ gene family (FOXO1, FOXO3, FOXO4 and FOXO6) act as versatile gene-specific transcription factors that regulate a variety of key cellular processes, such as cellular proliferation, stress response, stem cell maintenance and apoptosis [1]. One of these, FOXO3 (also referred to as FOXO3a, or FKHRL1 in the older literature) is of considerable interest in many therapeutically relevant areas, such as tumor therapy and longevity research [2,3].

Many aspects of FOXO3 activity are controlled by indirect growth factor signaling via phosphatidylinositol 3-kinase (PI3K), Akt and other kinases [4,5]. Post-translational phosphorylation events at specific sites of FOXO3 control the subcellular translocation, association with 14-3-3 proteins [6], DNA-binding properties and transcriptional activity [7]. Additional modifications, such as acetylation and ubiquitination, have also been detected [8]. Like essentially all eukaryotic gene-specific transcription factors, FOXO3 contains a structured DNA-binding domain (DBD) that allows the transcription factor to bind sequence-specifically to target sites in the genome containing a ‘forkhead response element’ (5′-(G/A)(T/C)(A/C)AA(C/T)A-3′) [9,10]. Many of the genes regulated by FOXO3 carry out anti-proliferative (cell cycle arrest) and pro-apoptotic functions (control of cell death), thus categorizing FOXO3 as a tumor suppressor [5,11,12].

**Figure 1:**
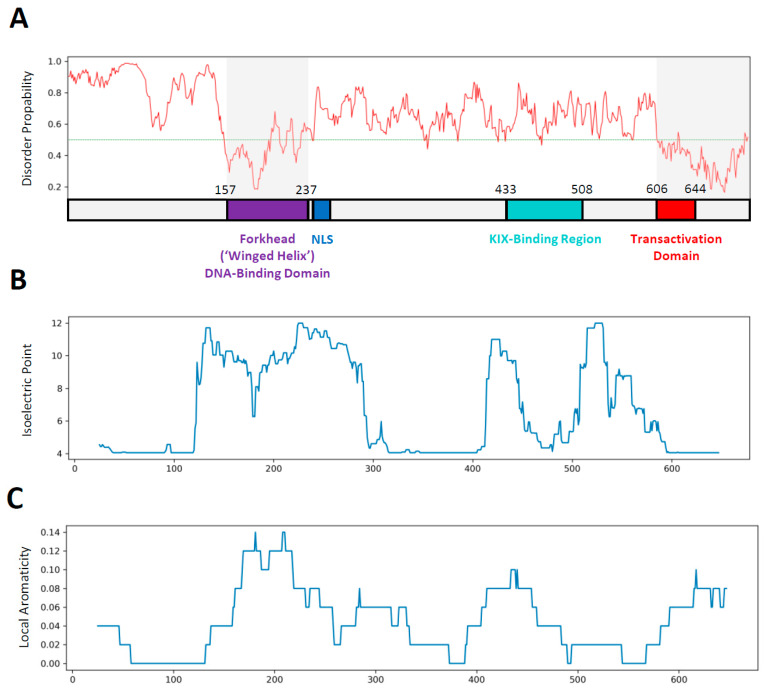
(**A**) Schematic diagram showing the distribution of known functional elements within the primary amino acid sequence of FOXO3 (annotation based on information associated with the NCBI reference sequence NP_963853.1). The start and end positions of several named elements (DNA-Binding Domain (purple), KIX-Binding Region (green) and the transcriptional Transactivation Domain are shown in the diagram). The start and end position of the nuclear localization sequence (NLS, dark blue) are 242 and 259, respectively. The top half of the panel shows the result of an IUPred2A analysis [14] of FOXO3. The red line indicates the probability of residues in that position to participate in a disordered structure. The green line indicates the cut-off point: residues below are considered structured. These values are congruent with the MobiDB-lite scores that predict an overall disorder of ~60% based on multiple criteria and methods (https://mobidb.org/ accessed on 18 May 2021; [15]). (**B**) Local isoelectric point analysis using a sliding window size of 25 residues. The calculated isoelectric value for each window is plotted according to the position of the center of the window. (**C**) Local aromaticity analysis using a sliding window size of 25 residues. The calculated hydrophobicity value (based on the proportion of large aromatic amino acids (Phe, Trp, Tyr) in the window) is plotted according to the position of the center of the window.

Inspection of the primary sequence of FOXO3 (673 amino acids) reveals a centrally located DBD flanked at both N- and C-termini by sequences predicted to be highly intrinsically disordered (Figure 1A; [13]). The primary amino acid sequence of these intrinsically disordered regions (IDRs) shows a highly uneven distribution of charged and hydrophobic amino acids (Figure 1B). Unlike other intrinsically disordered proteins (IDPs), the primary sequence of FOXO3 is highly conserved among vertebrates (Figure S1).

Outside the DBD, very little structural information is available. The C-terminal IDR is known to contain the KIX-Binding Region and Transactivation Domain (Figure 1A) that mediate transcriptional activation through their association with the CBP/p300 coactivator complex in a promiscuous manner [16,17]; these regions undergo a transition to α-helices upon binding to the coactivator surface [17].

The important biological roles of FOXO3, combined with the exceptionally high degree of predicted intrinsic disorder, make this transcription factor an interesting target for a more detailed structural characterization by computational simulations to gain further insights into such an unusual protein. Computational molecular dynamics (MD) simulations of intrinsically disordered proteins are challenging because many of the commonly used force-fields and water models have been derived from and optimized for stably folded proteins and are therefore prone to overestimate the structures of IDPs [18,19,20]. Computational simulation methods are, however, one of the most promising approaches for creating detailed models of IDRs and IDPs [21]; conventional strucural methods are either inapplicable (X-ray crystallography), or provide only population-averaged insights into secondary structure propensity (NMR; for example [22]), or overall molecular shape (SAXS; [21]).

Here, I present a series of atomistic models of full-length FOXO3 bound to DNA based on multiple independent simulations using an implicit MD simulation method, and another series of simulations of the FOXO3-DBD and part of the C-terminal IDR under fully explicit solvation conditions. In a previous study, both simulation conditions gave results that matched experimentally measured parameters well for the disordered N-terminal portion of c-MYC [23], thus showing that such simulation methods are capable of capturing essential structural features of IDPs. The results obtained provide plausible models for a spatially extended structure of FOXO3 when bound to DNA that will provide a better structural understanding to underpin future experimental approaches. A graphical method is introduced that is particularly helpful for visualizing differences in local conformation and folding propensities that will be of general use for analyzing MD trajectories of IDRs and IDPs.

## 2. Materials and Methods

### 2.1. Bioinformatic Analysis of Primary Sequence

The primary amino acid sequence of FOXO3 was obtained from the NCBI database (entry NP_963853.1). The sequence was analyzed using the IUPRED2 software [14] running locally. A sliding window analysis (window size 25 amino acids) of isoelectric point and aromaticity employed the Bio.SeqUtils.ProtParam modules from BioPython [24]. Window selection and sliding, data collection and plotting was carried out using custom-written Jupyter notebooks in Python3.

### 2.2. Creation of the Starting Structure for MD Simulations

The crystal structure of the FOXO3 DNA-binding domain bound to a DNA molecule containing a forkhead response element served as a partial starting structure (PDB# 2UZK). Additional PDB structures of the missing N-terminal (FOXO3^1−156^) and C-terminal (FOXO3^254−673^) IDR sequences were modeled by Markov Chain Monte Carlo simulation [25] and placed in position to link them covalently to the free termini of the DNA-binding domain. The DNA structure was extended using the web application ‘3DNA 2.0’ (http://web.x3dna.org/ accessed on 19 January 2021) with the ‘composite’ option. This maintains the original conformation of the DNA and contacts with the DNA-binding domain as described in the X-ray structure. The resulting composite structure (Figure S2) was parameterized with the Amber ff14SBonlysc forcefield (in conjunction with OL15 for DNA simulation, igb8 and the mbondi 3 atomic radii set) using the tLEaP program of the AMBER molecular modeling software package [26,27,28].

### 2.3. Implicit MD Simulations (gb8_md#1—gb8_md#10)

The simulations were carried out as described previously [23]. Briefly, after minimization, the production simulations were executed on GPUs [29]. Ten independent simulations were run, all starting from the same structure (Figure S2), for a total of 500 nanoseconds each.

### 2.4. Accelerated Molecular Dynamics of FOXO^120−530^ in TIP4P-D Water

The final structure generated by two of the implicit simulations (gb8_md#1 and gb8_md#2) provided the coordinates of the complete FOXO3 bound to DNA with the flexible linker region (FL#2) C-terminal to the DNA-binding domain in two alternative conformations (‘FL#2-A compact’ and ‘FL#2-A extended’). For preparing the partial structure, the coordinates of residues FOXO^1−119^ were replaced with an acetyl ‘cap’. The resulting structures were embedded in a cubic TIP4P-D [18] water box leaving a minimum of 20 Å between the molecule and the edge of the water box. After adjusting the ionic strength to 150 mM NaCl, the final systems containing either 597,606 atoms (Linker Conformation A) or 601,754 atoms (Linker Conformation B) were created. The models were minimized as described previously [23] and subjected to 20 ns of conventional molecular dynamics to obtain stable values for the dihedral and potential energies. These values were used to calculate the parameters for the accelerated MD (aMD) simulations [30], using an α-value of 0.2 as recommended in the Amber manual. Two sets of ten independent production simulations, each lasting 100 ns, were executed on GPUs for each of the two FL#2-A conformations [29].

### 2.5. Trajectory Visualization and Analysis Methods

The pdb files and MD trajectory data were visualized using Visual Molecular Dynamics (VMD, [31]). For various quantitative analyses (distances, root mean square deviation, secondary structures, etc.), various tools from cpptraj were used [32,33]. The data obtained were analyzed and plotted using custom-written Jupyter Python notebooks.

The pbsa program from the Amber Tools package [27] was employed for the Poisson-Boltzmann PBSA calculations. Standard settings were used except that the ionic strength (istrng) was set to 150 mM, temperature (pbtemp) to 310 K, and the atomic radii read from the prmtop file (radiopt = 0). Volumetric data files for the visualization of electrostatic potential and level set maps were created using the phiout function and graphically represented using VMD [31].

## 3. Results

### 3.1. FOXO3 Is Highly Conserved in Evolution, but Represents an Unusually Extensively Disordered IDP

**Figure 2:**
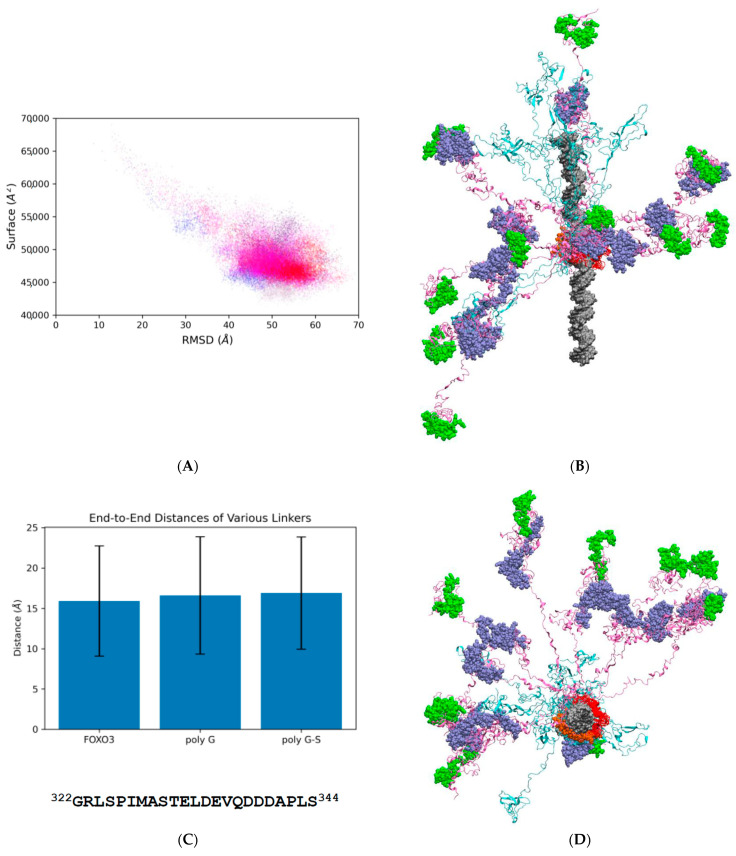
(**A**) Characterization of the molecular dynamics results using global variables (root mean square deviation [RMSD] and solvent accessible surface). The starting structure is located in the top left corner and the dots moving diagonally towards the lower right corner represent intermediate structures formed during the earliest stages of the simulations. All ten simulations finally generate structures that populate a dense space in the lower right corner. Each of the dots (different shades of red, blue, and purple represent different simulations) corresponds to the measurements from a single structure (‘snapshot’) sampled at 100 picosecond intervals in the trajectories (see Figure S3 for a Gaussian kernel density representation of this result). (**B**) Superimposition of snapshots representing the final structure (at 500 ns) from each of the 10 independent molecular dynamics simulations. The structures were aligned using the stably folded DNA binding motif (FOXO3^157−237^). The DNA is shown as a silver surface structure. The DNA-binding domain (DBD) is shown as a red cartoon and the nuclear localization sequence in orange van der Waals representation. The intrinsically disordered region (IDR) located N-terminally to the DBD is shown as a cyan cartoon model, and the IDR located C-terminally to the DBD as a pink cartoon model. The KIX-Binding Region is shown as blue van der Waals spheres, and the Transactivation Domain as green spheres. See also Video S1 for a rotating version of the structure for more details and to obtain a three-dimensional understanding of this figure. (**C**) Bar chart of the end-to-end distances of a segment of the flexible linker of FOXO3 (FOXO3^322-344^, sequence shown below) in comparison to a polypeptide of the same length consisting of glycine residues (‘polyG’) or alternating glycine-serine residues (‘polyG-S’) (**D**) Same structure as in (**B**), but viewed down the central axis of the DNA.

Application of the protein disorder prediction tool IUPred2 [14] to the primary sequence of human FOXO3 suggest the presence of extensively disordered regions located both N- and C-terminally of the structured DNA-binding motif (Figure 1A; [13]). Only the DNA-binding motif and the most C-terminal region, encompassing FOXO^600−673^ are predicted to take up a more folded conformation. The large size of the FOXO3 protein (673 amino acids), in conjunction with the large molecular radius of gyration predicted from its highly disordered nature, precludes atomistic molecular dynamics simulations of the complete molecule under explicitly solvated conditions for technical reasons. I therefore adopted a simulation strategy involving a generalized Born solvation mode (implicit gb8 solvation) MD [25]. The starting model contains extensively elongated N- and C-termini with little or no secondary structure elements to avoid biasing the simulations in any way (Figure S2). The starting model was used to initiate ten independent simulations, each lasting for 500 ns. Analysis of general system variables (root mean square deviation and solvent-accessible surface area) reveals that all simulations take a similar path through conformational space and converge on a series of structures within a relatively narrowly defined phase space (Figure 2A). Visual inspection of the final frames of all ten simulation shows, however, that this does not correspond to a single defined structure but represents a family of related structures with a highly variable composition of secondary structure elements and differently arranged N- and C-terminal sequences (Figure 2B,D; Video S1).

**Figure 3:**
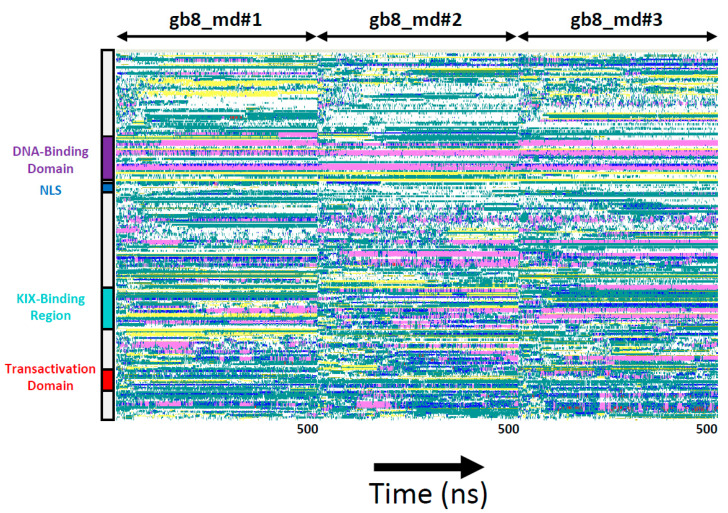
Distribution of secondary structure elements in molecular dynamics (MD) simulations. The distribution of secondary structure elements is shown for the first three MD simulations as representative examples (gb8_md#1, gb8_md#2, gb8_md#3). The vertical axis represents the primary amino acid positions, highlighting functionally relevant domains as colored boxes). The horizontal axis represents simulation time (1–500 nanoseconds (ns) for each of the three independent simulations). The data are represented in concatenated format to allow direct comparison between the different trajectories. The color codes represent the secondary structures formed in each simulation frame at each position of the primary amino acid sequence (pink, α-helix; dark blue, π-helix; yellow, extended conformation; cyan, turn; white, coil). Data visualized with VMD [34].

Further investigations revealed that in all ten simulations the α-helical elements and β-structure of the ‘winged helix’ DNA-binding domain remains fully structured as the domain remains bound to its DNA target site in a stable conformation. Both N- and C-terminal IDRs, however, display a variety of variable secondary structure elements (various types of helices, such π-, 3_10_ and α-helices, extended regions (β-sheets) and turns) of variable propensities (Figure 3 and Figure S4). It is of particular interest that in some simulations, a large proportion of extended structures are formed throughout the molecule (for example, gb8_md#1; Figure 3), whereas in other simulations, α- and π-helices predominate as the main secondary structure feature (for example, gb8_md#2 and gb8_md#3; Figure 3). A high proportion of bends and unstructured coils indicate a significantly unfolded state of FOXO3 in both N- and C-terminal IDRs. Once formed, secondary structures frequently only remain folded for a fraction of the simulation time before unfolding (and sometimes refolding) or interconverting to another type of secondary structure. Such fluidity in secondary structure content and stability is expected of an IDP.

Apart from local variation in secondary structures, it is also possible to discern a characteristic higher order of structural organization: both N- and C-terminal portions of FOXO3 are arranged as a mixture of extended and partially folded domains. Projection and superimposition of ten snapshots of the FOXO3/DNA complex at 500 nanosecond (ns) intervals (using the structurally stable DNA-binding domain to align all coordinates) reveals that the two regions adjacent to the DNA-binding domain stretch out and maintain a highly extended structure throughout most of the simulation time (Figure 2B,D; Video S1). I will refer to the flexible linkers located N- and C-terminally to the DNA-binding domain as FL#1 and FL#2, respectively.

**Figure 4:**
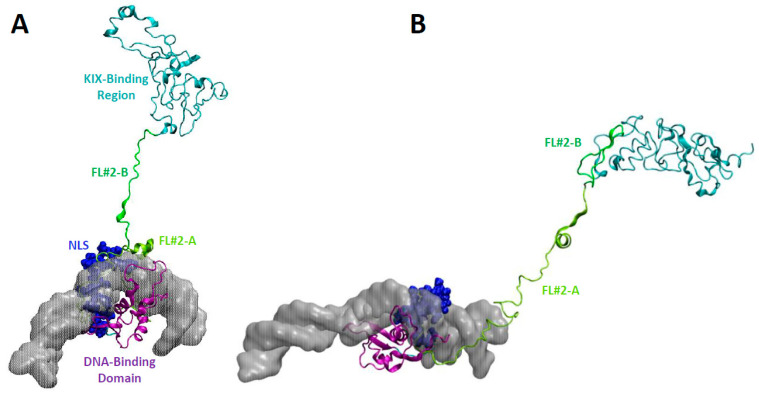
Stretched linker regions emanating from the DNA-binding and nuclear localization sequence (NLS). The DNA-binding domain is shown in a purple cartoon structure bound to DNA represented as a semitransparent surface. The NLS is shown in dark blue as van der Waals spheres. The first part of Flexible Linker #2 (FOXO3^260−321^; FL#2-A) is shown as a lime-green cartoon structure, the second part (FOXO3^322−344^; FL#2-B) in green. The KIX-Binding Region (FOXO3^433−508^) is represented as a cyan cartoon structure. (**A**) FL#2-A folds partially in a helical conformation and remains mostly associated with DNA (FL#2-A compact conformation). FL#2-B is highly extended. (**B**) FL#2-A is present in an extended conformation (FL#2-A extended conformation) and FL#2-B is mostly folded onto the KIX-Binding Region.

The unique structural properties of these linkers will be demonstrated using FL#2 as a specific example (Figure 4). This linker is organized in a bipartite manner with its N-terminal portion (FOXO3^260−321^; FL#2-A) and the C-terminal part (FOXO3^322−344^; FL#2-B) folding in different arrangements. FL#2-A remains in some simulations associated with the NLS and DNA and displays partial helical secondary structure (‘FL#2-A compact’; Figure 4A). In other snapshots, FL#2-A is extended and emanates from the DNA-binding domain/NLS without contacts with DNA (‘FL#2-A extended’; Figure 4B). FL#2-B also exists in alternative conformations: extended or associated with the more C-terminally located KIX-Binding Region. Accordingly, the length of FL#2 varies between 40 and 70 Å, with an average end-to-end distance of around 55 Å.

**Figure 5:**
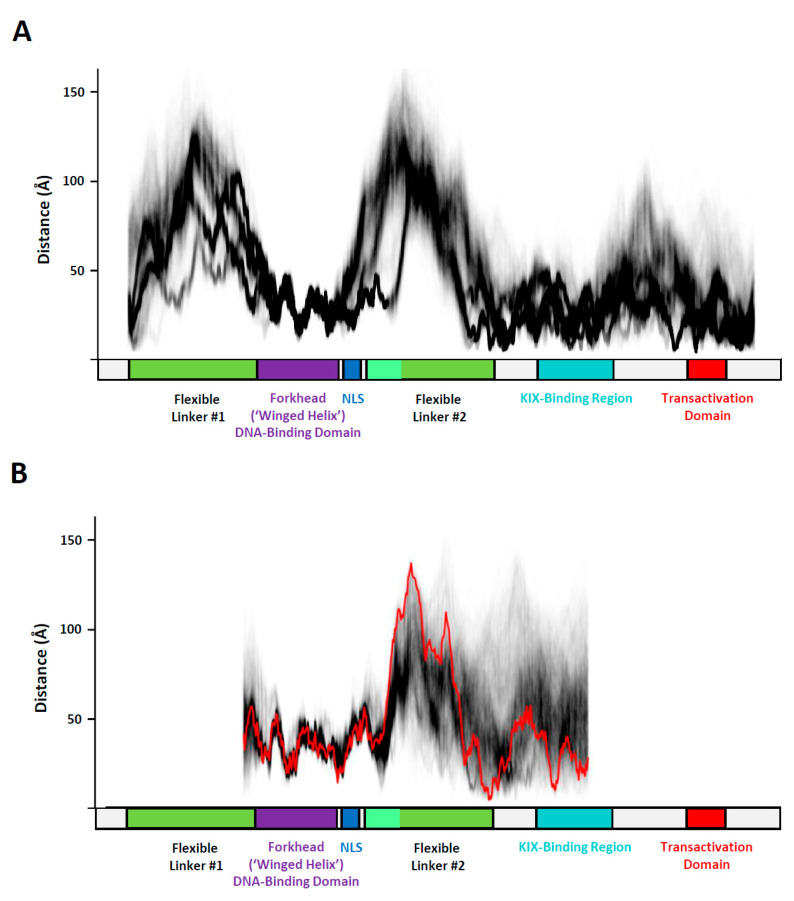

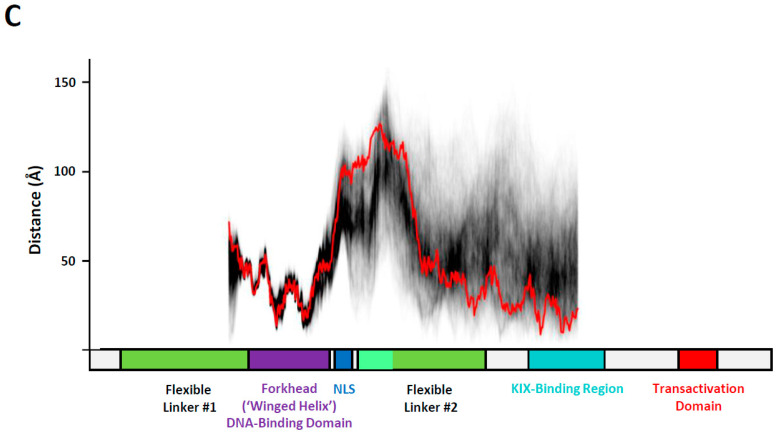
Local compaction plots (LCPs) of FOXO3 molecular dynamics (MD) simulation data. (**A**) LCP analysis of the FOXO3 MD simulation data. The sliding window size for the intramolecular distance measurements is fixed at 75 amino acids. The distance (in Å) between the residues at position 1 and 75 of the window is determined using cpptraj (AMBER simulation package; [32]) for all snapshots of the trajectories. The window is then moved to a new position shifted by one residue. The FOXO3 DNA-binding domain (residues 157 to 237, shown as a purple box) is stably folded and therefore all distance measurements within (and immediately adjacent to it) are quite constant, short (<50 Å) and superimposable on each other to form a dense black line. In contrast, the ‘Flexible Linkers’ (FL) are distinctly recognizable in the LCP because of the large distances (50–150 Å) identified in the sliding 75 amino acid windows and their more diffuse appearance due to the presence of alternative conformations. (**B**) LCP of the aMD simulation data of FOXO3^120−530^ using the final frame of gb8_md#1 containing FL#2-A in a compact conformation. The LCP trace of the starting structure is shown in red and the traces of the aMD simulations reflecting 100 ns in black. (**C**) LCP of the aMD simulation data of FOXO3 120–530 using the final frame of gb8_md#1 containing FL#2-A in an extended conformation. The LCP trace of the starting structure is shown in red and the traces of the aMD simulations reflecting 100 ns in black.

The variability in the extension of FL#2 can be visualized effectively in a ‘local compaction plot’ (LCP). LCPs provide qualitative and quantitative insights into this phenomenon based on the complete trajectory data available. Creation of an LCP is conceptually straightforward: the intramolecular distances between residues spaced apart by a fixed span on the primary amino acids (a ‘window’) are measured in all trajectory snapshots and plotted. In the examples shown below, a window size of 75 amino acids was chosen to provide a clear visual distinction between folded and unfolded regions. The distance values obtained between the two residues at the beginning and end positions for each window in every frame are plotted along the x-axis. Subsequently, the window is moved by one residue and the pairwise distance measurements are repeated as described above. The ‘sliding window’ approach is repeated until the end of the sequence represented in the MD simulation is reached. While this creates a massive amount of data (with (tens of) thousands of frames typically present in a MD trajectory and hundreds of sliding window positions), the output can be visualized in a graphical format that is easily interpretable by the human eye by plotting each data point transparently. This allows all measurements to be represented according to their frequency. Frequent measurements are represented as a darker image, whereas alternative conformations occurring at lesser frequency are still shown albeit as a lighter representation. This way of presenting local conformational data is of special value for studying the conformational diversity of MD simulations of IDPs. In the case of FOXO3, the LCP method identifies distinctly the stably folded DNA-binding domain (FOXO3^157−237^) from the remainder of the protein (Figure 5).

The FOXO3 DBD (FOXO^157−237^) is highly folded, and therefore all distance measurements within (and immediately adjacent to it) are invariant, short (<50 Å) and superimposable on each other to form a densely black line. In contrast, the ‘Flexible Linkers’ (FL-1 and FL-2) are distinctly recognizable in the LCP because of the large distances (50–150 Å) identified in the sliding 75 amino acid windows and their more diffuse appearance due to conformational heterogeneity. As pointed out earlier, for FL#2 the simulations reveal at least two distinct patterns, that either include or exclude the NLS sequence located immediately C-terminal to the DBD; whereas in some simulations this structure is still stably structured, in other simulations the NLS becomes part of a more extended FL2 (Figure 4 and Figure 5A). Interestingly, the KIX-Binding Region and C-terminal region containing the Transactivation Domain (TAD) show evidence of notable folding (distances mostly <50–60 Å). The folding is, however, heterogeneous (multiple preferred conformations and containing a subset of unfolded structures as revealed by the diffuse grey lines). This heterogeneity shows that they are still intrinsically disordered, although to a lesser extent than the more flexibly structured linkers FL#1 and FL#2. This example illustrates the simplicity of the concept underlying LCP, while highlighting its uses in interpreting large, complex data sets of MD simulations of IDPs.

**Figure 6:**
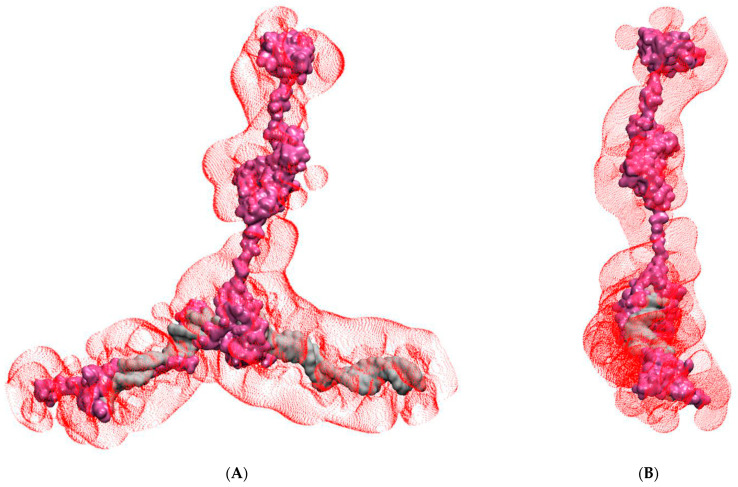
Electrostatic surface of FOXO3 bound to DNA. (**A**) Frontal view of the isoelectric surface representation of the FOXO3-DNA complex. The structure of the FOXO3-DNA complex is shown in surface representation (DNA silver, FOXO3 purple). The red dotted surface represents the isosurface with an isovalue of 0.02. (**B**) Same as in (**A**) but rotated by 90° to allow viewing of the complex along the DNA axis.

Many IDPs contain unfolded polypeptide chains, but these tend to bend and fold into more compact shapes (‘random coils’) without displaying a comparable degree of rigidity. It was therefore of special interest to look more closely at the structural properties of the rigid linker regions FL-1 and FL-2 present in FOXO3 to determine the structural basis of their enhanced propensity to form extended conformations. Inspection of the amino acid sequence (for example, FL#2B; FOXO3^322−344^, Figure 2C) revealed no unusual features other than an excess of negatively charged amino acid residues. Simulation of FL#2B on its own with identical implicit solvation conditions applied to the intact DNA-FOXO3 complex revealed no distinct rigidity; the distribution of the end-to-end distance of FL#2B was statistically indistinguishable from highly flexible polypeptides of the same length consisting of poly-glycine (poly-G) or alternating glycine and serine residues (poly-G/S) (Figure 2C). It is therefore evident that the extended nature of FL#2B is not encoded directly by its primary amino acid sequence. If we consider the FL regions of FOXO3 to be highly flexible, other physical forces must be responsible for maintaining their extended conformations observed in the simulations of the intact FOXO3/DNA complex. Of all the non-covalent forces determining molecular conformations, only electrostatic forces are capable of long-distance action. A Poisson-Boltzmann surface area (PBSA) plot indeed reveals a significant net negative charge covering the DNA and FOXO3 bound to it (Figure 6). The linkers share this excess negative charge property, thus providing a physicochemical explanation for the extended conformations of FOXO3.

### 3.2. Confirmation of Electrostatic Repulsion Effect in Explicit Solvent Models

The portion of the molecule comprising the DNA, DBD and initial portion of the C-terminal domain represents one of the clearest examples of the electrostatic repulsion effect (Figure 5A). The use of implicit solvation models is generally reliable and has previously provided good results for other intrinsically disordered domains (such as c-MYC; [23]), but they may display a tendency to generate structures that are folded more compactly than expected. An artefactually high compaction could result in a denser clustering of charges and therefore could be responsible for the electrostatic repulsion effect observed in the implicit solvation simulations of FOXO3. I therefore set up another series of simulations with explicitly solvated models using TIP4P-D optimized specifically for the simulation of IDPs [18]. A portion of FOXO3 lacking the N-terminal IDR (FOXO3^120−530^), but including the DNA-binding domain, the nuclear localization sequence, FL#2 and the more densely folded KIX Binding Region (Figure 1A) was embedded in a TIP4P-D water box and subjected to ten independent accelerated molecular dynamics (aMD) simulations, each lasting 100 nanoseconds. Two sets of simulations, using the final structures of either gb8_md#1 or gb8_md#2—containing FL#2-A in either a compact or extended conformation, respectively, were tested. aMD simulations are thought to represent molecular motions occurring at a time scale that is two to three orders of magnitude higher than the actual simulation time, so the conformational changes observed most likely cover at least one microsecond [30]. TIP4P-D is a water model that was developed specifically for simulating IDPs and produces molecular models that confirm closely to experimentally observed biophysical measurements, such as radius of gyration [18,20,23]. Analysis of the aMD results with the LCP method in an identical manner to the one employed previously for the data from the implicit MD trajectories reveals a comparable outcome (Figure 5B,C). As observed previously, the DBD and NLS remain distinctly folded, and FL-2 extends to values in the 80–150 Å range and is thus comparable to the results represented by the starting model (gb8_md#1 and gb8_md#2 after 500 ns, respectively). In the aMD simulations, the region containing the KIX-Binding Region is folded less compactly than with the implicit simulations (as indicated by the larger intramolecular distance measurements in that region; Figure 5B,C), which is in line with the expectations from simulations run the TIP4P-D water model. It can be concluded that the electrostatic repulsion model identified in the implicit simulations is compatible with results from explicitly solvated simulation regimes and therefore reflects a genuine property of the molecular system studied.

### 3.3. Conformational Variability in the KIX-Binding Region and Transactivation Domain

**Figure 7:**
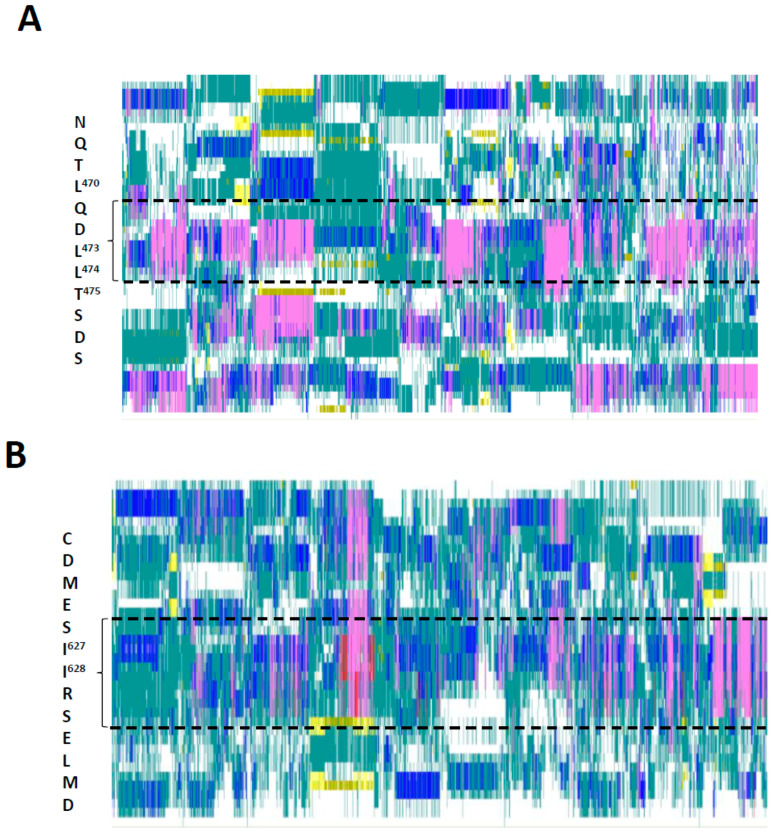
Secondary structures of the KIX-Binding Region and Transactivation Domain (TAD) formed in ten implicit solvation simulations (gb8_md#1—gb8_md#10), each representing 500 nanoseconds. The positions and sequences of α-helical regions shown are based on experimentally determined locations of KIX-binding motifs [17]. (**A**) The region (residues 451 to 500) surrounding the α-helix participating in binding to the KIX-domain of the CBP/p300 coactivator complex is shown. The position and sequence of the interaction helix (FOXO^467−478^) binding to the KIX domain directly is highlighted. (**B**) The region (residues 606 to 644) surrounding another α-helix participating in binding to the KIX-domain of the CBP/p300 coactivator complex is shown. The position and sequence of the interaction helix (FOXO^622−634^) binding to the KIX domain directly is highlighted. The color codes represent the secondary structures formed in each simulation frame at each position of the primary amino acid sequence (pink, α-helix; dark blue, π-helix; red, 3_10_ helix; yellow, extended conformation; cyan, turn; white, coil). Data visualized with VMD [34].

The activation function of FOXO3 depends on two distinct motifs found in the C-terminal IDR, the KIX-Binding Region (FOXO^433−508^) and Transactivation Domain (‘TAD’; FOXO^606−644^) (Figure 1A). Although named distinctly, both regions appear to carry out their transactivation functions in a similar manner involving the (simultaneous) binding of their α-helices (FOXO^467−478^ and FOXO^622−634^, respectively) within surface grooves of the KIX domain in the CBP/p300 coactivator. This is presumably achieved by the flexible linker that separates the two motifs within the FOXO3 primary sequence looping out from the coactivator surface [17]. The simulation data presented here are in full agreement with this model. Analysis of the secondary structures formed in the KIX-Binding Region in FOXO3—in the absence of coactivator binding—shows that both interaction motifs are predisposed to form helical conformations, including α-, π- and 3_10_ helices (Figure 7). Especially the centrally located hydrophobic residues known to play a key structural and functional role in these interactions (L^470^, L^473^, L^474^ and I^627^, I^628^) are in regions with particularly high helical propensity. Such structural predispositions of intrinsically disordered activation domain motifs to form helical structures in the absence of their binding partner is a well-known property of IDPs (‘templated folding’; [35,36]) and has been detected in other well-studied model systems of TADs [37,38]. Evidence for a flexible linker connecting the KIX-Binding Region and TAD is also evident from the LCP (Figure 5A).

## 4. Discussion

FOXO3 is an intriguing protein. The high degree of evolutionary conservation of the primary amino acid sequences, especially among vertebrates (Figure S1), is reminiscent of the level of conservation found in structured proteins, where the identities of individual amino acids determine secondary and tertiary structure elements much more rigidly than they do in IDPs. Bioinformatic prediction tools suggest, however, that FOXO3 is mostly disordered outside the centrally located DNA-binding motif (MobiDB [15]; Figure 1A). FOXO3 is therefore either a highly unusual IDP that does not conform to the rules apparently emerging from many other IDPs or contains a higher proportion of sequence-determined structural elements than is evident from the sequence-based bioinformatic analyses. To address this question, a series of fully atomistic MD simulations of a complete FOXO3-DNA complex is presented here. The results confirm the extensive intrinsically disordered nature of the protein outside the DBD as demonstrated by the highly variable and fluctuating secondary structure elements in the N- and C-terminal IDRs (Figure 3 and Figure 5). Interestingly, a systematic analysis of the higher-order structural arrangement shows substantial variation in local folding densities. The DBD is flanked on both sides by highly extended linkers, FL#1 and FL#2, and the more compactly folded KIX-Binding Region and TAD are also separated by a linker of variable length (Figure 2B,D, Figure 5A–C; Video S1). These linkers lack any intrinsic ‘stiffness’ as determined by their primary sequences, but the domains they are separating are negatively charged and thus repel each other and stretch the connecting unstructured polypeptide sequences (Figure 6). This excess negative charge includes the surface of the DBD that contains a high proportion of positively charged amino acids (Figure 1C), but these are employed primarily for electrostatic binding to DNA. The extended overall structure of the IDRs fully exposes the known functional domains, especially the KIX-Binding Region and TAD near the C-terminus, in a highly flexible manner to protein–protein interactions with the CBP/p300 coactivator complex [17] by providing a large capture radius (‘fly casting’ mechanism [39]; Video S1). Additionally, the flexibility of FOXO3 may facilitate the subsequent formation of phase separated transcription complexes in conjunction with other components of the transcriptional machinery [40,41]. FOXO3 is a target for several post-translational modifications, especially phosphorylation. Specific phosphorylation at 20 different positions of FOXO3 (mostly in the C-terminal IDR) by various kinases has been documented. Current functional explanations for these phosphorylation events focus on their roles in subcellular localization and regulating transcription activity [42,43,44]. The electrostatic repulsion mechanism controlling the spatial extension of FOXO3 proposed above suggests that at least some of these phosphorylations may have (additional) conformational effects be controlling intramolecular distances between different parts of FOXO3.

## 5. Conclusions

The computational simulation of IDPs, such as gene-specific transcription factors, is still technically challenging. While the models obtained may not be perfectly accurate in all aspects, it is clear that some of the most fundamental aspects of FOXO3—the variable extent of folding of the different intrinsically disordered domains and their mutual electrostatic repulsion—is a very robust, yet previously unrecognized property that is revealed reliably through MD simulations.

## Data Availability

The complete molecular dynamics simulation data and/or copies of the analysis Jupyter notebooks are available from the author on request.

## Supplementary Materials

The following are available online at https://www.mdpi.com/article/10.3390/biom11060856/s1, Figure S1: Sequence alignment of vertebrate and invertebrate orthologs of FOX03, Figure S2: Starting model of FOXO3 bound to DNA, Figure S3: Gaussian kernel density estimation of the phase space determined by root mean square (RMSD) and solvent accessible surface area measurements from the ten independent implicit simulations over 500 ns simulation time. Figure S4: Frequency of secondary structure elements across ten independent molecular dynamics simulations (gb8_md#1—gb8_md#10, 500 nanoseconds each), Video S1: FOXO3-DNA_complex.
