# Supplementary material for: Molecular Dynamics Simulations of Human FOXO3 Reveal Intrinsically Disordered Regions Spread Spatially by Intramolecular Electrostatic Repulsion
Source: Biomolecules. 2021 Jun 8;11(6):856. doi: 10.3390/biom11060856 (PMC8228108; doi:10.3390/biom11060856)
Supplement: Supplementary file 1 [file biomolecules-11-00856-s001.zip › Supplementary_files/Supplementary Information 2021-04-15.pdf]

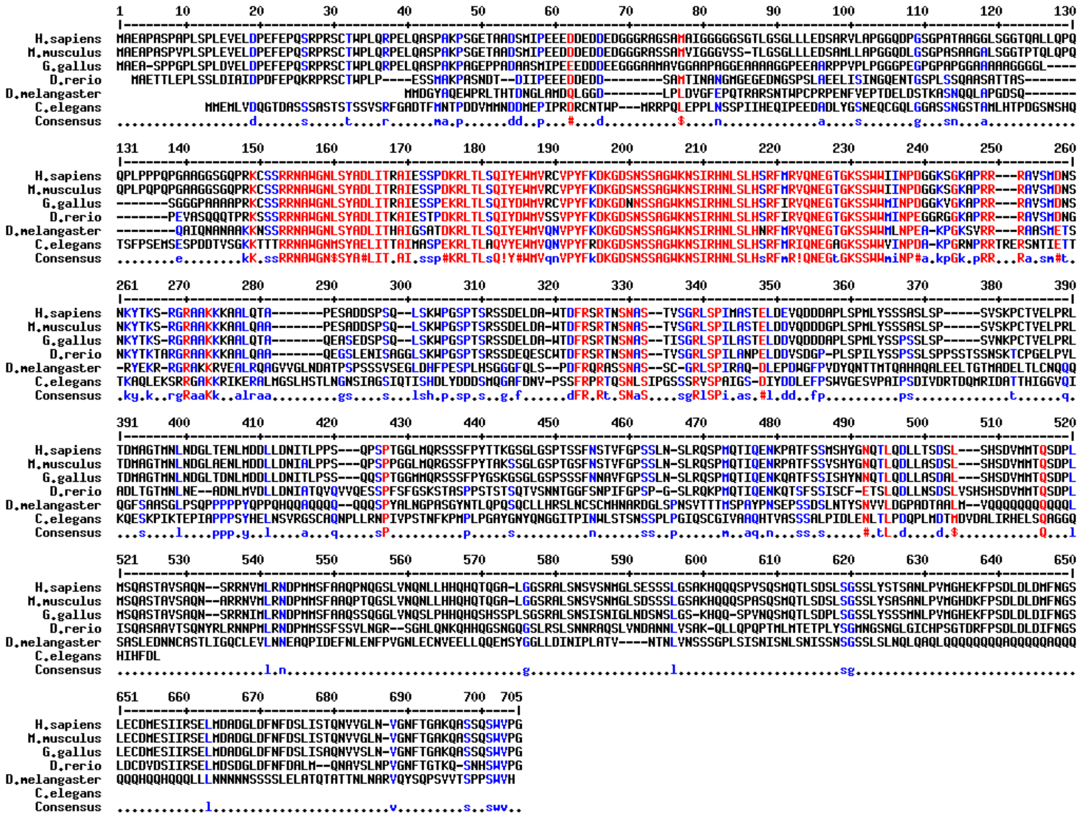

Figure S1. Sequence alignment of vertebrate and invertebrate orthologs of FOX03. The sequences shown and their corresponding NCBI access codes are *Homo sapiens* (NP_001446), *Mus musculus* (mouse; NP_001363896), *Gallus gallus* (chicken; XP_001234496), *Danio rerio* (zebrafish; NP_571160), *Drosophila melanogaster* (fruit fly; NP_001262557), and *Caenorhabditis elegans* (nematode worm; NP_001364784).

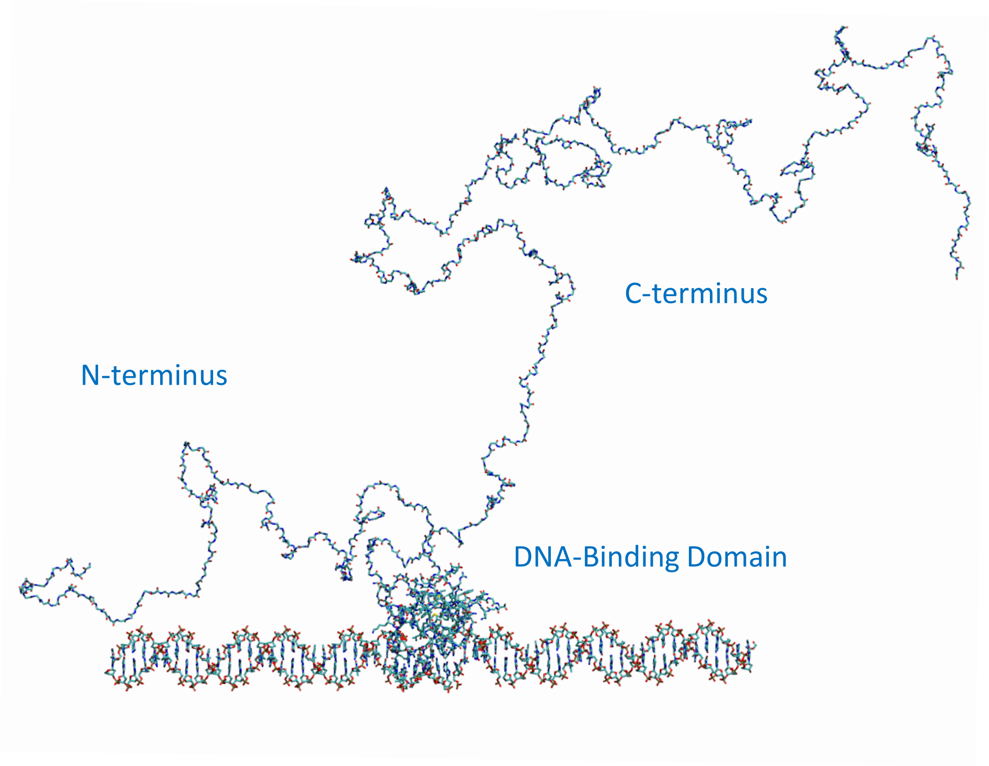

Figure S2. Starting model of FOXO3 bound to DNA. Extended structures representing the N-and C-termini were added to the crystal structure of the FOXO3 DNA-binding domain bound to DNA (PDB# 2UZK). The added N- and C-termini contain structures of the polypeptide chain devoid of specific secondary structure elements to avoid any undue structural bias in the subsequent molecular dynamics simulations. Similarly, the ends of the DNA molecule in PDB# 2UZK were extended with random sequence to generate a longer DNA molecule.

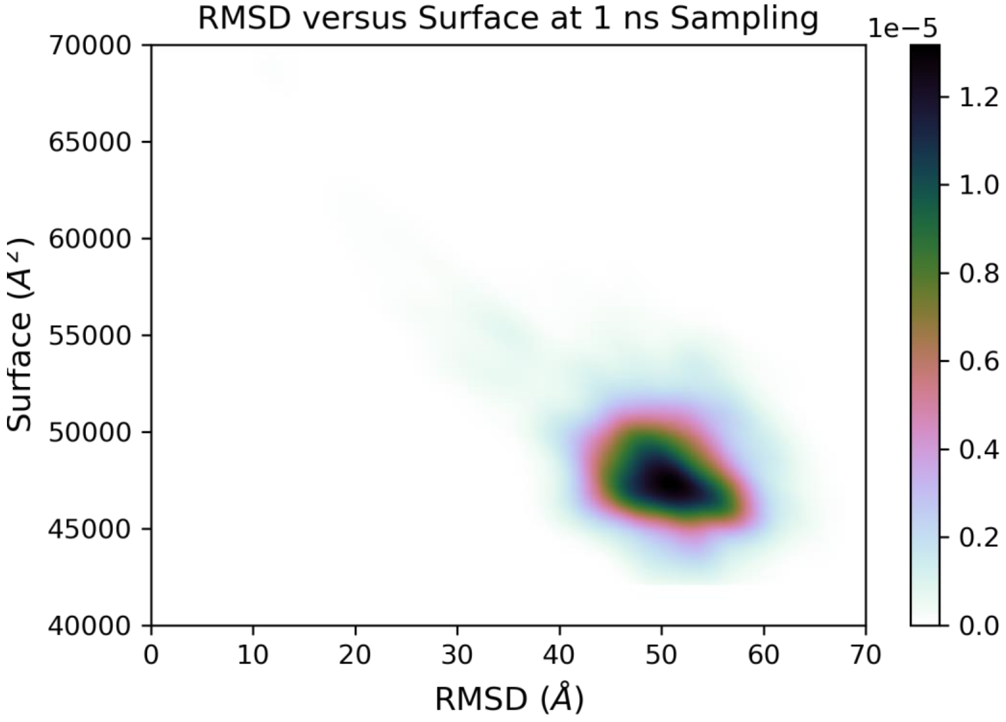

Figure S3. Gaussian kernel density estimation of the phase space determined by root mean square (RMSD) and solvent accessible surface area measurements from the ten independent implicit simulations over 500 ns simulation time. The scale is indicated by color bar ('cubehelix' color scheme) on the right hand side.

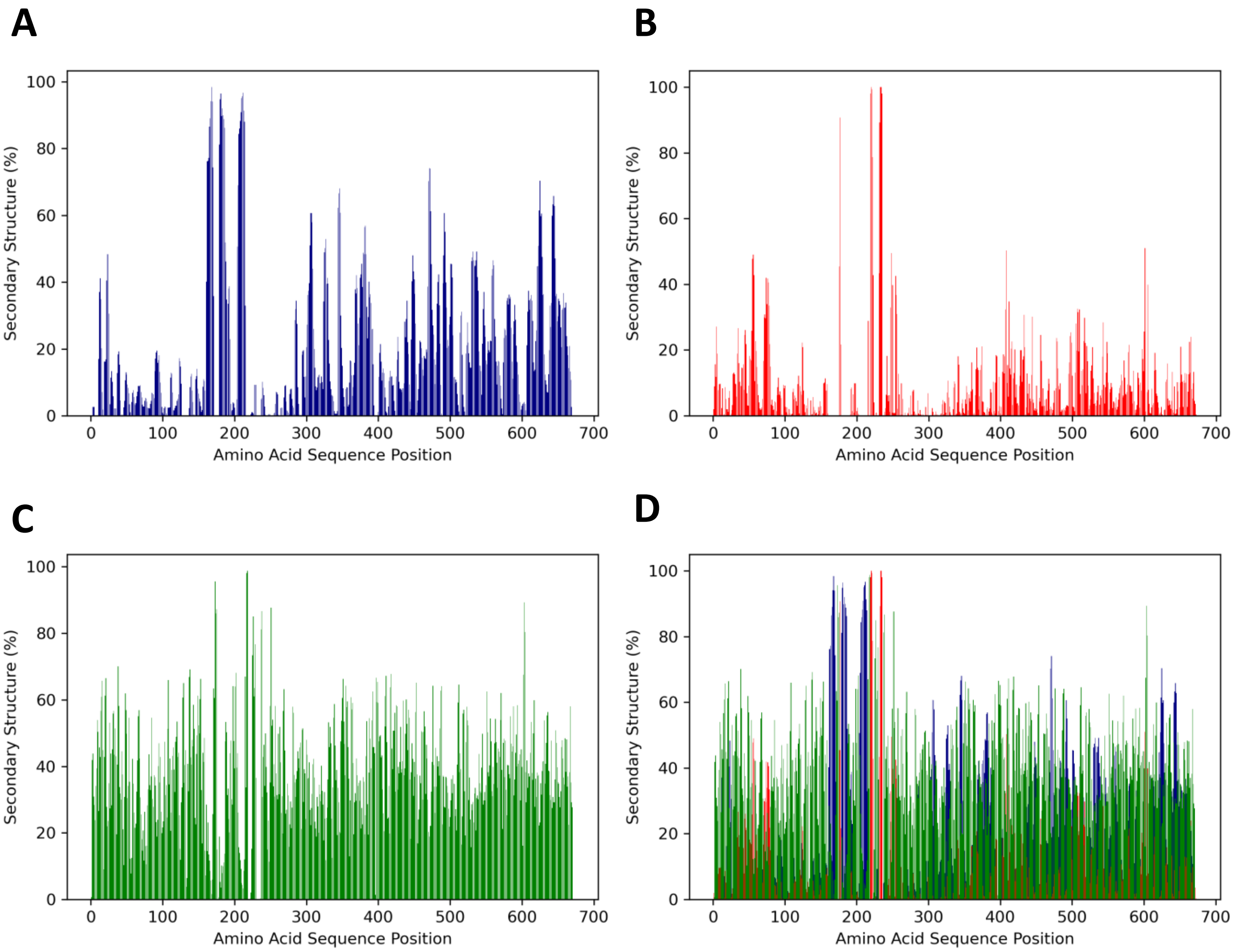

Figure S4. Frequency of secondary structure elements across ten independent molecular dynamics simulations (gb8_md#1 - gb8_md#10, 500 nanoseconds each). The frequency of formation of specific structures is shown on the vertical axis. The amino acid position across the primary amino acid sequence is shown on the horizontal axis. (**A**) Frequency of helical structures (combined values for α-, π- and 3_{10}-helices). (**B**) Frequency of extended structures (combined values for parallel- and anti-β sheets). (**C**) Frequency of extended structures (combined values for bends and turns). (**D**) Frequency of all identified secondary structure elements (combined data as shown in (A), (B) and (C)). The DNA-binding domain emerges distinctly as the most stable structure with essentially 100% stability.
